# Analysis of the Chemical Composition of the Essential Oil of *Polygonum minus* Huds. Using Two-Dimensional Gas Chromatography-Time-of-Flight Mass Spectrometry (GC-TOF MS)

**DOI:** 10.3390/molecules15107006

**Published:** 2010-10-12

**Authors:** Syarul Nataqain Baharum, Hamidun Bunawan, Ma’aruf Abd. Ghani, Wan Aida Wan Mustapha, Normah Mohd Noor

**Affiliations:** 1 Institute of Systems Biology, Universiti Kebangsaan Malaysia, 43600 Bangi, Selangor, Malaysia; 2 School of Chemical Sciences and Food Technology, Faculty of Science and Technology, Universiti Kebangsaan Malaysia, 43600 Bangi, Selangor, Malaysia; 3 School of Biosciences and Biotechnology, Faculty of Science and Technology, Universiti Kebangsaan Malaysia, 43600 Bangi, Selangor, Malaysia

**Keywords:** comprehensive two-dimensional gas chromatography, volatile oil, TOF MS, *Polygonum minus* Huds, GC-MS, retention indices

## Abstract

The essential oil in leaves of *Polygonum minus* Huds., a local aromatic plant, were identified by a pipeline of gas chromatography (GC) techniques coupled with mass-spectrometry (MS), flame ionization detector (FID) and two dimensional gas chromatography time of flight mass spectrometry (GC×GC–TOF MS). A total of 48 compounds with a good match and high probability values were identified using this technique. Meanwhile, 42 compounds were successfully identified in this study using GC-MS, a significantly larger number than in previous studies. GC-FID was used in determining the retention indices of chemical components in *P. minus* essential oil. The result also showed the efficiency and reliability were greatly improved when chemometric methods and retention indices were used in identification and quantification of chemical components in plant essential oil.

## 1. Introduction

*Polygonum minus* Huds, commonly known as kesum, is widely used in Malaysian cooking, and several traditional practices utilise the leaves and stems of this plant [1]. Kesum is an aromatic plant that produces high levels of essential oil (72.54%) containing aliphatic aldehydes [2]. Yaacob [2] identified decanal (24.36%) and dodecanal (48.18%) as the two dominant aldehydes that contribute to the flavour of kesum. Apart from decanal and dodecanal, Yaacob also found that kesum contains 1-decanol (2.49%), 1-dodecanol (2.44%), undecanal (1.77%), tetradecanal (1.42%), 1-undecanol (1.41%), nonanal (0.86%), 1-nonanol (0.76%), and β-caryophyllene (0.18%). As a result, kesum is believed to have great potential as a natural source of aliphatic aldehydes, which could be useful as food additives and in the perfume industry.

With the development of botanical drugs, including traditional herbal medicines, analysis of their bioactive components is becoming more popular. Many botanical drugs have bioactive components in their essential oils, so characterization of plant essential oils it is an important and meaningful task. Gas chromatography (GC) or gas chromatography-mass spectroscopy (GC-MS) are used almost exclusively for the qualitative analysis of the volatiles [3].

Natural essential oils are usually mixtures of terpenoids (mainly monoterpenoids and sesquiterpenoids), aromatic compounds and aliphatic compounds. As mass spectra of these compounds are usually very similar, peak identification often becomes very difficult and sometimes impossible. In order to address the qualitative determination of composition of complex samples by GC-MS and to increase the reliability of the analytical results, it is necessary to utilize retention indices identities [4].

Meanwhile, comprehensive, two-dimensional gas chromatography (GC×GC) also has been extensively applied in the essential oil study [5]. This technique has also been successfully used in the industrial analysis of plant materials to improve component separation and identification. In addition, an analysis of *Artemisia annua* L. volatile oils using multi-dimensional gas chromatography has indicated that this technique can achieve the complete separation of a wide range of terpenes [6]. 

The objective of this study was to demonstrate different gas chromatography approaches to analyse the composition of the essential oils of kesum, with the hope that the improved component separation and identification would allow for a determination of unidentified minor components that may strongly influence the overall quality of the oil.

## 2. Results and Discussion

Using GC–MS, Yaacob detected only 10 components in kesum essential oil [2], with decanal and dodecanal being identified as marker compounds. According to the literature [7], a similarity and reverse number greater than 800 and a probability value greater than 1,000 indicate that an acquired mass spectrum is a good match with a library spectrum. Further identification information, including retention time, similarity, reverse, and probability values, greatly increases the reliability of this analysis. For a comparison study, we have also applied kesum essential oil on GC-MS. In our analysis we found 42 significant compounds in kesum essential oil, significantly more than the number reported by Yaacob [2], and all these compounds had similarity indexes or reverse similarities greater than 800 (Table 1). The retention indices for each compound are also presented in Table 1.

**Table 1:** Compounds identified in the essential oil of kesum using gas chromatography mass spectrometry (GC-MS).

No.	Rt	Compound	*Retention Indices	Formula	Similarity	R.Match	Probability(%)	Content (%)
1	5.066	Hexanal	803	C_6_H_12_O	917	948	50	0.05
2	6.922	1-Hexanol	868	C_6_H_14_O	888	895	39.1	0.09
3	8.932	α-Pinene	932	C_10_H_16_	953	954	17.2	0.39
4	14.979	Undecane	1101	C_11_H_24_	947	948	51.5	0.41
5	15.154	Nonanal	1106	C_9_H_18_O	916	933	78.2	0.15
6	17.581	1-Nonanol	1173	C_9_H_20_O	887	944	45.1	0.05
7	18.857	Decanal	1209	C_10_H_20_O	950	950	74.3	16.263
8	21.113	1-Decanol	1274	C_10_H_22_O	951	959	31.9	12.68
9	21.484	Isobornyl acetate	1285	C_12_H_20_O_2_	858	879	18	2.39
10	22.289	Undecanal	1308	C_11_H_22_O	950	959	60.7	0.14
11	24.412	*n*-Decanoic acid	1373	C_10_H_20_O_2_	918	920	57.7	0.52
12	24.495	α-Cubebene	1376	C_15_H_24_	757	823	10.2	0.37
13	24.9	Xanthorrhizol	1388	C_15_H_22_O	876	923	59.9	0.1
14	25.709	Dodecanal	1413	C_12_H_24_O	955	972	56	43.47
15	25.926	(*E*)-Caryophyllene	1420	C_15_H_24_	947	948	18.4	3.83
16	26.347	*trans*-α-Bergamotene	1434	C_15_H_24_	938	955	54	0.49
17	26.63	α-Bisabolol	1443	C_15_H_26_O	818	836	11.4	0.06
18	26.947	Farnesene	1453	C_15_H_24_	907	908	31.2	0.18
19	27.039	α-Caryophyllene	1456	C_15_H_24_	939	942	54.6	1.02
20	27.64	1-Dodecanol	1475	C_12_H_26_O	821	945	18	1.19
21	27.719	β-Himachalene	1478	C_15_H_24_	787	850	42.8	0.48
22	27.907	α-Selinene	1484	C_15_H_24_	851	860	6.6	0.15
23	28.073	Valencene	1489	C_15_H_24_	828	896	7.8	0.32
24	28.294	δ -Cadinine	1496	C_15_H_24_	835	854	10.7	0.19
25	28.507	Alloaromadendrene	1503	C_15_H_24_	754	817	4.8	0.06
26	28.69	α-Curcumene	1510	C_15_H_22_	861	904	17.1	0.18
27	28.974	(-)-α-Panasinsene	1519	C_15_H_24_	886	889	27.4	0.27
28	29.145	cis -Lanceol	1525	C_15_H_24_O	824	845	34.4	0.27
29	29.708	Farnesol	1544	C_15_H_26_O	819	827	7.3	0.14
30	30.029	Humulene	1555	C_15_H_24_	752	805	15.5	0.13
31	30.213	Nerolidol	1561	C_15_H_26_O	823	902	15.4	0.24
32	30.288	Dodecanoic acid	1564	C_12_H_24_O_2_	847	854	49	0.23
33	30.826	β-Caryophyllene oxide	1582	C_15_H_24_O	883	885	50.1	0.35
34	31.526	*trans*-α- (*Z*)-Bergamotol	1606	C_15_H_24_O	856	865	71.2	0.13
35	31.739	Tetradecanal	1614	C_14_H_28_O	958	980	44.3	0.1
36	32.064	Alloaromadendrene oxide-(1)	1625	C_15_H_24_O	791	821	12.2	0.31
37	32.294	*trans*- Longipinocarveol	1634	C_15_H_24_O	828	851	8.1	0.28
38	32.448	Neoisolongifolene, 8-bromo-	1639	C_15_H_23_Br	790	849	11.7	3.09
39	35.117	*iso*-Caryophyllene	1737	C_15_H_24_	842	901	10.2	0.08
40	35.997	Drimenol	1770	C_15_H_26_O	930	930	77.6	2.01
41	40.471	Drimenin	1941	C_15_H_22_O_2_	835	938	81.8	0.28
42	44.25	Phytol	-	C_20_H_40_O	891	903	45.5	0.13

* Experimentally determined Kováts retention indices.

We also carried out GC-MS analysis by using multiple internal standards for quantification of compounds. The standard curve of standard mixtures was used to determine concentration of selected compounds in kesum essential oil. We found that the α-pinene content in kesum was 0.02 mg/mL. Meanwhile, drimenol was found at a concentration of 0.79 mg/mL, along with humulene (0.047 mg/mL), caryophyllene (0.031 mg/mL) and farnesol (0.030 mg/mL).

GCxGC-TOF MS analysis showed 48 significant compounds in kesum essential oil, six compounds more than detected by our GC-MS and all of these compounds had similarity values greater than 800 (Table 2). 

**Table 2:** Compounds identified in the essential oil of kesum using two-dimensional gas chromatography time-of-flight mass spectrometry GC×GC–TOF MS.

No	t^1^_R_ (s)	t^2^_R_ (s)	Name	Formula	Similarity	Reverse	Probability	Content (%)^a^
1	440	1.960	2-Hexenal	C_6_H_10_O	861	861	5910	0.001
2	445	1.740	*cis*-3-Hexenal	C_6_H_10_O	882	882	6575	0.022
3	510	0.950	Nonane	C_9_H_2_0	907	907	4995	0.062
4	575	0.755	3-Carene	C_10_H_16_	829	837	1783	1.202
5	605	0.895	Camphene	C_10_H_16_	903	903	3220	0.009
6	670	0.890	Sabinene	C_10_H_16_	886	887	4388	0.013
7	975	0.905	Undecane	C_11_H_24_	926	937	5688	2.286
8	995	1.500	Nonanal	C_9_H_18_O	896	896	8416	0.010
9	1270	1.055	Cyclodecanol	C_10_H_20_O	839	839	1534	5.691
10	1275	1.185	Decanal	C_10_H_20_O	951	951	8419	23.121
11	1445	1.115	2-Butyltetrahydrofuran	C_8_H_16_O	925	925	4928	0.004
12	1445	1.260	1-Decanol	C_10_H_22_O	938	938	3392	2.090
13	1450	1.860	1-Cyclopropylpentane	C_8_H_16_	850	865	1220	0.005
14	1465	1.085	Isobornyl formate	C_11_H_18_O_2_	877	883	1980	0.071
16	1520	1.135	Undecanal	C_11_H_22_O	969	973	7142	0.990
17	1680	0.930	α-Copaene	C_15_H_24_	865	865	4693	0.024
18	1695	1.650	Octylcyclopropane	C_11_H_22_	908	908	2274	0.001
19	1720	1.055	(*Z,E*)-α-Farnesene	C_15_H_24_	839	862	4954	0.928
20	1770	0.980	α -Cedrene	C_15_H_24_	842	842	2894	0.012
21	1790	1.090	1-Dodecanal	C_12_H_24_O	942	942	5140	4.785
22	1800	1.275	Dodecanal	C_12_H_24_O	963	974	6783	38.635
23	1805	0.895	(*E*)-β-Caryophyllene	C_15_H_24_	898	898	4747	0.212
24	1830	0.875	α-Bergamotene	C_15_H_24_	943	952	4035	0.801
25	1845	0.910	γ -Gurjunene	C_15_H_24_	878	884	3364	0.095
26	1870	0.920	α-Humulene	C_15_H_24_	898	898	8426	2.293
27	1885	0.945	*trans*-β-Farnesene	C_15_H_24_	836	852	4383	0.907
28	1930	1.215	1-Dodecanol	C_12_H_26_O	936	936	1769	1.380
29	1940	0.860	2-Isopropenyl-4a,8-dimethyl-1,2,3,4,4a,5, 6,7-Octahydro-naphthalene	C_15_H_24_	890	894	1897	0.697
30	1940	1.230	α-Curcumene	C_15_H_22_	882	882	9172	0.080
31	1955	0.870	Valencene	C_15_H_24_	869	896	1392	0.806
32	1985	0.950	Alloaromadendrene	C_15_H_24_	859	860	1321	0.039
33	2000	1.150	β-Bisabolene	C_15_H_24_	816	816	3899	0.014
34	2005	1.195	α-Zingiberene	C_15_H_24_	796	827	2546	0.013
35	2020	0.870	α-Panasinsene	C_15_H_24_	888	888	4332	0.563
36	2035	0.945	δ-Cadinene	C_15_H_24_	870	875	5111	0.025
37	2095	1.215	Patchulane	C_15_H_26_	804	807	1237	0.004
38	2130	1.320	Nerolidol	C_15_H_26_O	872	873	6205	0.075
39	2170	0.990	Caryophyllene oxide	C_15_H_24_O	914	915	6752	1.513
40	2200	1.140	Ocimene	C_10_H_16_	808	871	2319	0.055
41	2235	1.090	Tetradecanal	C_14_H_28_O	875	931	2259	1.056
42	2280	0.955	dehydro- Cyclolongifolene oxide	C_15_H_22_O	810	812	3756	0.544
43	2280	1.130	Acoradiene	C_15_H_24_	845	886	1125	0.079
44	2280	1.155	1,3,6,10-Dodeca-tetraene	C_15_H_24_	807	837	989	0.117
45	2290	1.260	4,4-Dimethyltetra-cyclo[6.3.2.0(2,5).0(1,8)]tridecan-9-ol	C_15_H_24_O	847	847	4371	0.122
46	2550	1.205	Drimenol	C_15_H_26_O	930	930	8162	0.574
47	3200	1.465	Phytol	C_20_H_40_O	801	814	3655	0.003
48	3400	1.260	Hexadecanal	C_16_H_32_O	805	805	1312	0.004

^a^ t^1^_R_ and t^2^_R_ retention times of peaks on first and second dimension, respectively; ^b^ Content is the peak volume percentage of compounds in the essential oil sample.

Compounds found both in GC-MS and GC×GC-TOF MS are shown in Table 3. Out of 42 compounds found in GC-MS analysis, only 21 compounds were also found in GC×GC–TOF MS. This may be due to the less sensitivity of GC-MS compared to GC×GC–TOF MS. The relative concentrations of several classes of volatile compounds in kesum are shown in Table 4.

**Table 3:** The essential oil compounds found both in GC-MS and GC×GC–TOF MS.

No	Name	Formula
1	Undecane	C_11_H_24_
2	Nonanal	C_9_H_18_O
3	Decanal	C_10_H_20_O
4	1-Decanol	C_10_H_22_O
5	Undecanal	C_11_H_22_O
6	Dodecanal	C_12_H_24_O
7	(*E*)-β-Caryophyllene	C_15_H_24_
8	*trans*-α-Bergamotene	C_15_H_24_
9	α-Humulene	C_15_H_24_
10	*trans*-β-Farnesene	C_15_H_24_
11	1-Dodecanol	C_12_H_26_O
12	α-Curcumene	C_15_H_22_
13	Valencene	C_15_H_24_
14	Alloaromadendrene	C_15_H_24_
15	α-Panasinsene	C_15_H_24_
16	δ-Cadinene	C_15_H_24_
17	Nerolidol	C_15_H_26_O
18	Caryophyllene oxide	C_15_H_24_O
19	Tetradecanal	C_14_H_28_O
20	Drimenol	C_15_H_26_O
21	Phytol	C_20_H_40_O

**Table 4:** Relative concentrations of several classes of volatile compounds in kesum.

Chemical class of volatile compound	% Relative area
Esters	0.071
Furans	0.004
Alcohols	9.857
Aldehydes	68.624
Hydrocarbons and terpenes	13.489

**Figure 1:**
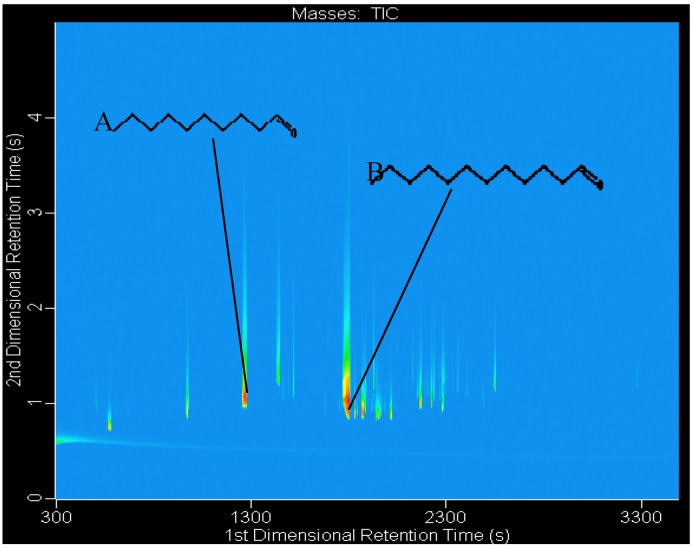
2D-GC chromatogram contour plots of *Polygonum minus* Huds. volatile oil and structural pictures of (A) decanal and (B) dodecanal as the main constituents.

In GC×GC–TOF MS analysis, the 48 identified compounds were classified into groups, including one ester, one furan, five alcohols, nine aldehydes and 32 hydrocarbons. Therefore, the majority of the components found in the kesum volatile oil were terpene compounds. The number of terpenes found is far more than that reported by Yaacob [2], where only β-caryophyllene was observed and identified. Although decanal and dodecanal have been identified as the dominant components in the oil (Figure 1), we believe that the terpene group may also contribute strongly to the flavour of kesum. The presence of this group is shown in Table 2 and Table 4, and the significant components that exhibited a good match index with a compound in the NIST MS database are listed. This study demonstrates that GC×GC–TOF MS is a powerful separation and identification tool that allows for the identification of a much larger number of complex volatile oil components.

**Figure 2:**
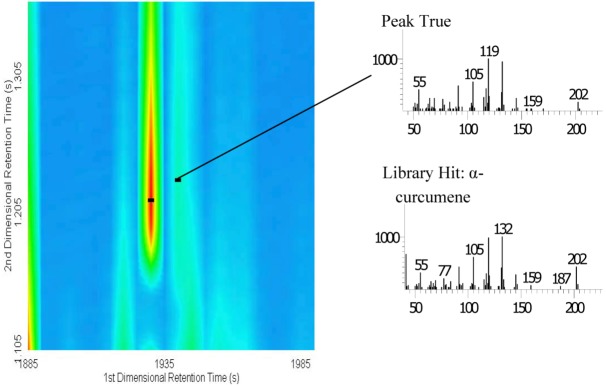
Details of the GC×GC contour plot chromatogram of α-curcumene, peak spectra of the sample, and peak spectra reported in the NIST library.

A probability value greater than 9,000 reflects that the mass spectrum is highly unique and could be the source of flavour and bioactive compounds in a mixture, identifying a compound that may be valuable for further pharmaceutical research. Based on our GC×GC–TOF MS result, we found that only α-curcumene had a probability value above 9,000. The sesqueterpenoid α-curcumene is produced as a major component in the essential oil of several plants, including *Curcuma longa,* and serves as an insect repellent and insect-feeding deterrent [8]. In our study, we tentatively identified α-curcumene in the essential oil of kesum (Figure 2).

## 3. Experimental

### 3.1. Plant material

Fresh leaves of kesum were collected in January 2009 from the Genting Highland, Pahang, Malaysia (3° 25′ 22.43″ N, 101° 47′ 32.38″ E). Voucher specimens were deposited in the Herbarium of the Faculty of Science and Technology, Universiti Kebangsaan Malaysia, Bangi, Malaysia (UKMB).

### 3.2. Extraction procedure

Two hundred and fifty grams of kesum were subjected to hydrodistillation for 8 hours using a Clevenger-type apparatus [9]. The essential oils were collected over water, separated, dried over anhydrous sodium sulphate, and stored in the dark at 4 ºC prior to GC-FID, GC-MS and GC×GC–TOF MS analysis.

### 3.3. GC×GC–TOF MS analysis

The comprehensive two-dimensional gas chromatograph system employed consisted of an Agilent 6890N GC equipped with a flame ionisation detector (Agilent, Palo Alto, CA, USA) and filled with a cold-jet modulator KT-2007 retrofit prototype (Zoex Corporation, Lincoln, NE, USA). A time-of-flight mass spectrometer (Pegasus 4D, LECO Corporation, St. Joseph, MI, USA), equipped with an Agilent 6890N GC, was used to acquire mass spectral data. The MS parameters included a 70-eV electron impact ionisation value and a maximum spectral acquisition rate of 500 spectra per second. Two capillary columns were used, connected by a universal press-tight connector, and were installed in the same oven. The column sets used are listed in Table 5. 

**Table 5:** Features of the GC×GC column sets.

	Column 1	Column 2
Length (m)	30	1
Diameter (mm)	0.25	0.25
Stationary phase	Rtx-5MS	DB-wax
Film thickness (μm)	0.10	0.25
Corporation	Restek Corporation, Bellefonte, PA	J&W Scientific, Folsom, CA

Ultra-high purity (99.999%) helium was used in constant pressure mode as the carrier gas. The inlet pressure was 72.4 psi. An Agilent 7683B auto sampler was used to inject 1 μL of the sample with a splitless injector into the inlet of column 1 at 250 ºC. Column 1 was held at 45 ºC for 2 min, and then, the temperature was increased at a rate of 3 ºC/min until the column reached a final temperature of 200 ºC. Column 2 was set to be 15 ºC warmer than column 1. The mass spectrometer was operated at an acquisition rate of 50 spectrals. No mass spectra were collected during the first 3 min of the solvent delay. The modulation period was 5 s. The transfer line and the ion source temperature were 250 ºC and 200 ºC, respectively. The detector voltage was 1600 V, and the electron energy was -70 V. Mass spectra were collected from 50–400 m/z. The pressure inside the flight tube was approximately 1^-7^ torr. In the identification analysis, LECO^®^ Software Version 3.34 was used to find all of the peaks in the raw chromatograms. The parameters, such as the similarity, reverse, and probability values of peaks identified through a library search using NIST/EPA/NIH Version 2.0, were combined into a single peak table.

### 3.4. GC-MS analysis

The essential oils were analysed using a Clarus 600 GC-MS system (Perkin Elmer, USA). The compounds were separated on 30 m × 0.25 mm × 0.25 μm Elite-5MS column (Perkin Elmer, USA). The column temperature was increased from 40 ºC to 220 ºC at a rate of 4 ºC/min; injector temperature, 250 ºC; injection volume, 1 μL; transfer temperature, 280 ºC. MS parameters were as follows: EI mode, with ionization voltage 70 eV, ion source temperature, 180 ºC; scan range, 50-600 Da. The peaks were tentatively identified based on library search using NIST and Wiley Registry 8 Edition. The identities of some components were confirmed by both mass spectral and retention data of the authentic chemicals obtained under identical GC-MS conditions. Internal standards were applied and concentration of selected compounds was determined based on standard calibration curve.

### 3.5. GC-FID analysis and n-Alkane standard solutions

In order to perform Kováts indices, the essential oil were analysed using a Hewlett Packard 5890 system GC-FID (Hewlett Packard, Palo Alto, CA, USA). The compounds were separated on 30 m × 0.25 mm × 0.1 µm DB-5HT column. The GC program was the same as those used for GC-MS analysis. n-alkane standard solutions C_8_-C_20_ (mixture no. 04070) and C_21_-C_40_ (mixture no. 04071) were purchased from Fluka Chemica. Retention indices of essential oil compounds was carried out according to standard method of Kováts Indices to support the identification of the compounds.

## 4. Conclusions

GC-MS can perform much more reliable qualitative and quantitative analysis of complex essential oils samples. Meanwhile, GC-FID eventually was a very basic chromatograph technique, but provides us more information on retention indices that are crucial in analytical chemistry. However, GC×GC-TOF MS system for the analysis of kesum essential oil identified five times more compounds than those reported from a previous study using GC-MS, and we found that the majority of these compounds were terpenes. We believe that the 10 major components in the essential oil of kesum detected by previous research exclude many minor components that should not be ignored, as they also strongly contribute to the overall qualities of the essential oil. 
